# Biosynthesis of Silver Nanoparticles Using *Ligustrum Ovalifolium* Fruits and Their Cytotoxic Effects

**DOI:** 10.3390/nano8080627

**Published:** 2018-08-18

**Authors:** Bianca Moldovan, Vladislav Sincari, Maria Perde-Schrepler, Luminita David

**Affiliations:** 1 Research Center for Advanced Chemical Analysis, Instrumentation and Chemometrics (ANALYTICA), Faculty of Chemistry and Chemical Engineering, Babeş-Bolyai University, 11 Arany Janos Street, Cluj-Napoca 400028, Romania; bianca@chem.ubbcluj.ro; 2 Faculty of Chemistry and Chemical Engineering, Babeş-Bolyai University, 11 Arany Janos Street, Cluj-Napoca 400028, Romania; shinkar.vlad@mail.ru; 3 “Ion Chiricuta” Oncology Institute, 34–36 Republicii Street, Cluj-Napoca 400015, Romania; pmariaida@yahoo.com

**Keywords:** phytosynthesis, silver nanoparticles, *Ligustrum ovalifolium* L., cytotoxic activity, ovarian carcinoma cells

## Abstract

The present study reports for the first time the efficacy of bioactive compounds from *Ligustrum ovalifolium* L. fruit extract as reducing and capping agents of silver nanoparticles (AgNPs), developing a green, zero energetic, cost effective and simple synthesis method of AgNPs. The obtained nanoparticles were characterized by UV-Vis spectroscopy, transmission electron microscopy (TEM), X-ray diffraction (XRD) and Fourier Transform Infrared spectroscopy (FTIR), confirming that nanoparticles were crystalline in nature, spherical in shape, with an average size of 7 nm. The FTIR spectroscopy analysis demonstrated that the AgNPs were capped and stabilized by bioactive molecules from the fruit extract. The cytotoxicity of the biosynthesized AgNPs was in vitro evaluated against ovarian carcinoma cells and there were found to be effective at low concentration levels.

## 1. Introduction

Nanostructures of noble metals were lately immensely investigated due to their remarkable physical and chemical properties. The beneficial effects of silver salts have been noticed since antiquity. Reducing the particle size of materials is an efficient and reliable tool to improve their biocompatibility. Nanoparticles can be synthesized by several ways, such as physical, chemical or biological methods. Silver nanoparticles can be obtained by various chemical and photochemical reduction reactions, by thermal decomposition, by electrochemical methods, radiation or sonochemical assisted synthesis [1]. All these processes are efficient techniques to synthesize silver nanoparticles but they also have some drawbacks. The physical and chemical processes are expensive and use hazardous chemicals which may generate important environmental problems and can require a great deal of energy [2]. The as synthesized silver nanoparticles are chemically contaminated and require an advanced purification especially when they are intended to be used for medical applications. The biological methods are environmental friendly, cost effective and easily scaled up for large scale synthesis of nanoparticles and involve microorganisms, enzymes or plant extracts [3,4,5]. Various recent studies demonstrated the efficacy of fruit extracts such as *Acacia nilotica*, *Phoenix dactylifera*, *Tamarindus indica, Sambucus nigra, Licium barbarum* in the synthesis of silver nanoparticles [6,7,8,9]. The phytochemical compounds present in fruits such as flavonoids, carotenoids, aldehydes, ketones, proteins and carboxylic acids may act as bioreducing agents for Ag ions to silver nanoparticles. Metal nanoparticles obtained by phytomediated green synthetic methods combine the biological effects of metal and bioactive molecules present in the plant extract which are responsible for the reduction and stabilization of the nanoparticles, so they can be used as reliable tools in the field of nanomedicine [7,10,11,12]. Plant mediated synthesized nanoparticles have also the advantage of being safer for biomedical purposes as microbe or chemical mediated synthesized nanoparticles [13,14].

In the recent years, several biomedical applications have been reported for silver nanoparticles [15,16,17]. Since the ancient times, silver has been used in wound healing and in the 19th century its antimicrobial activity was established, this being the most well-known and exploited biological application of silver nanoparticles. Apart their antibacterial activity, AgNPs have been also proved as efficient antifungal and antivirucidal agents (inhibit HIV, Takaribe virus, hepatitis B, A/H1N1 virus) [16,18]. Recent publications reported the potential therapeutic applications of silver nanoparticles in cancer and inflammatory diseases [4,6,10,16].

*Ligustrum ovalifolium* L. is commonly called California privet or garden privet, is an ornamental semi-evergreen shrub original from East Asia, widely cultivated as ornamental plant. *Ligustrum* (privet) fruits are known to contain phenolic acids, flavonoids and triterpenoids, responsible for their antihyperglycemic, anticarcinogenic effect and immunomodulatory activity [3,19,20,21]. 

Traditional Chinese medicine uses privet fruits as tonic for liver and kidneys [17]. Modern medicine recorded the extract of these fruits to possess immunomodulatory, anti-inflammatory, antitumor and anti-ageing effects [22]. *Ligustrum lucidum* fruits exhibit antiproliferative activity against lung, breast, liver, pancreatic and colorectal carcinoma cells [23,24,25]. 

Ovarian carcinoma is one of the leading primary causes of cancer-related fatality in women [26]. Therefore, finding new therapeutic agents to fight against the proliferation of these carcinoma cells is of great concern.

The objective of the present work was to develop a phytomediated green synthesis method of silver nanoparticles, without using any environmental deleterious chemical reducing or capping agents such as sodium borohydride, Tollens reagent, *N*,*N*-dimethyl formamide and polyvinyl alcohol (PVA) [27], by exploiting the antioxidant effects of compounds present in the *Ligustrum ovalifolium* L. fruit extract and to investigate their cytotoxicity against A2780 ovarian carcinoma cells. 

## 2. Materials and Methods

### 2.1. Reagents

Cell titre blue reagent was purchased from Promega (Darmstadt, Germany). Cell lines and all other chemicals and reagents were purchased from Sigma-Aldrich (Darmstadt, Germany) and were of analytical purity. 

### 2.2. Preparation of the Extract

Garden privet fruits were harvested in September 2017 from Cluj-Napoca, Romania. To 2.5 g of fresh milled fruits, 50 mL of distilled water were added and the mixture was stirred for 1 h at room temperature and then filtered.

### 2.3. Determination of Total Phenolic Content

The total phenolic content of the garden privet fruits extract was colorimetrically determined using the Folin-Ciocalteu method [28]. Aliquots of 250 μL extract were transferred into a test tube (each analysis was conducted in triplicate) and then mixed with 1.5 mL of Folin-Ciocalteu reagent. The samples were incubated in the dark for 5 minutes and then 1.2 mL of sodium carbonate solution (0.7 M) was added and thoroughly mixed. After standing 2 hours at room temperature in the dark, the absorbance of the resulting blue solutions was measured at 765 nm, using a double beam UV-Vis spectrophotometer (Perkin-Elmer Lambda 25, PerkinElmer Inc., Waltham, MA, USA). The total phenolic content was calculated and expressed as gallic acid equivalents (mg GAE/L) using a calibration curve of the gallic acid standard solution (0–100 μg/mL). 

### 2.4. Determination of Antioxidant Activity

The free radical scavenging activity was determined using the ABTS (2,2′-azinobis-3-ethyl-benzthiazino-6-sulphonic acid) method [29]. The ABTS^+^⋅ stock solution was obtained by mixing equal volumes of 7mM ABTS solution with an oxidant agent (2.45 mM potassium persulfate solution). The stock solution was diluted to give an absorbance between 0.6–0.8 at 734 nm. Aliquots of 50 μL fruit extract were added to 3 mL diluted ABTS^+^⋅ solution and the absorbencies of the samples were red at 734 nm exactly after 15 minutes. The antioxidant activity was calculated using a calibration curve of Trolox standard (0–400 μmol/L Trolox) and expressed as μM Trolox equivalents.

### 2.5. Synthesis of AgNPs

The green synthesis of silver nanoparticles was achieved by adding 5 mL of *Ligustrum ovalifolium* L. fruit extract to 25 mL 1 mM aqueous silver nitrate solution under stirring at room temperature for the bioreduction of Ag^+^ ions to Ag^0^. The effect of the pH of the reaction medium on the silver nanoparticles formation and characteristics was investigated over a pH range from 6 to 10. The influence of the pH value on the formation of AgNPs was spectrophotometrically monitored. The pH of the resulting solution was adjusted by adding 1% aqueous NaOH solution. The rapid formation of a yellowish-brown colour indicated the reduction of the silver ions. After 3 hours under constant stirring, the resulting solutions were centrifuged at 10,000 rpm for 15 minutes. The separated nanoparticles were washed with double distilled water and dried. Three mg solid AgNPs were obtained.

The influence of the ratio of silver ions to the fruits extract which act as reducing agent was investigated at optimized pH value. Different amounts of plant extract were added to 25 mL 1mM silver nitrate solution in order to achieve the desired ratios (1:1; 1:3; 1:7; 1:10; 1:12 and 1:15 respectively).

### 2.6. Characterization of the AgNPs

The formation and the stability of silver nanoparticles were spectrophotometrically monitored, using a Perkin Elmer Lambda 25 UV-Vis spectrophotometer. The morphological characteristics of the synthesized nanoparticles were carried out using transmission electron microscopy (H-7650 120 kV Automatic transmission electron microscopy (TEM), Hitachi, Tokyo, Japan). In order to identify the biomolecules responsible for capping and stabilizing the silver nanoparticles, these were analysed by Fourier transform infrared spectroscopy (FTIR) using a Bruker Vector 22 FT-IR spectrometer, Bruker, Dresden, Germany). The obtained AgNPs were subjected to X-Ray diffraction analysis, data being recorded on a D8 Advance X-ray diffractometer with CuK_α_1 radiation.

The thermogravimetric analysis (TGA) of the biosynthesized AgNPs obtained using *Ligustrum* fruit extract was realised in nitrogen atmosphere using a thermogravimetric analyser (SDT Q600, TA Instruments, New Castle, DE, USA) in a temperature range 40–1000 °C, at a heating rate of 10°/min.

A Malvern Zetasizer Nanoseries compact scattering spectrometer (Malvern instruments Ltd., Worcestershire, UK) was used to determine the zeta potential of the synthesized silver nanoparticles. The hydrodynamic diameter of particles was determined in aqueous solution also by the Zetasizer.

### 2.7. Cytotoxic Activity

The synthesized AgNPs were tested on two cell lines: A2780 and A2780-Cis (cisplatin resistant), two human ovarian carcinoma cell lines. The cells were maintained in specific media: Roswell Park Memorial Institute (RPMI), supplemented with 10% foetal calf serum, glutamine and antibiotics. The cells were kept at 37 °C in a humid incubator with 5% CO_2_.

The AgNPs cytotoxicity was evaluate by a fluorometric method, using cell titre blue. This is a homogeneous fluorometric method which estimates the number of viable cells which maintain their metabolic capacity to reduce resazurin (dark blue colour) to resofurin (pink-purple). Resofurin is highly fluorescent and can be quantified with a fluorescence plate reader at 579 nm extinction and 584 nm emission.

Cells were plated in 96-well plates at a density of 20,000 cells/well. The cells were treated with different concentrations of AgNPs solutions: 180; 90; 45; 22.5; 9; 4.5; 2.25; 0.9; 0.01 µg/mL. The cells were also treated with serial dilutions of the *Ligustrum ovalifolium* L. extract. From the initial concentration of the extract: 190 µg GAE/mL, the following concentrations were obtained: 170; 142.5; 95; 47.5; 19; 9.5; 4.75; 1.9 µg/mL. As a positive control, the cytotoxicity of cisplatin on the ovarian carcinoma cell lines was tested. After 24h incubation, 20 µL cell titre blue reagent was added to 100 µL culture medium in each well. After 1h incubation the fluorescence was recorded. At each concentration, the surviving fraction was calculated as fluorescence of the sample/fluorescence of control (non-treated cells) × 100. Each experiment was done in triplicate.

Statistical analysis was performed using GraphPad Prism 5 software (San Diego, CA, USA). Inhibitory concentration (IC50) values were calculated using the non-linear regression. Comparisons were made using one-way ANOVA, considering *p* < 0.05 as criteria of significance. 

## 3. Results and Discussion

### 3.1. Synthesis and Characterization of Silver Nanoparticles

Oleuropein, luteolin and ligstroside are the main flavonoids present in privet fruits [30], compounds which confer these fruits a high antioxidant capacity and make them suitable candidates for the reduction of metallic ions to metallic nanoparticles. The antioxidant capacity of the privet fruit extract was determined using the ABTS assay and was found to be 340 μM Trolox. The total phenolic content of the extract, assessed by the Folin Ciocalteu method, was 190 μg GAE/mL.

**Figure 1:**
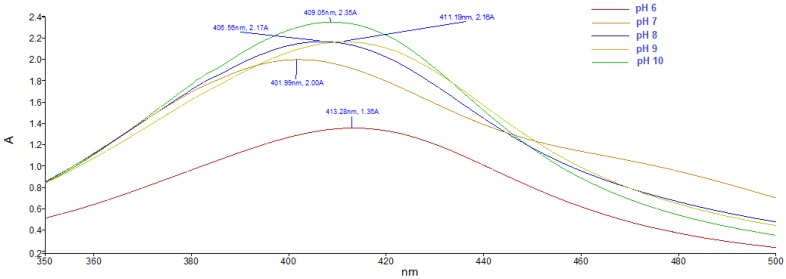
UV-Vis spectra of silver nanoparticles at different pH values.

The bioactive molecules present in the *Ligustrum ovalifolium* L. fruit extract served as reducing and capping agents for the synthesis and stabilization of silver nanoparticles. The colour change of the synthesis mixture to a yellowish brown colour, due to the surface plasmon resonance (SPR) of the silver nanoparticles in the solution, indicated the bioreduction of the silver ions [31]. The UV-Vis spectra of the silver nanoparticles obtained at different pH values are presented in Figure 1. The SPR peaks of AgNPs appeared between 402 and 413 nm, in accordance to the characteristic SPR band of silver nanoparticles, typically in the range of 400–500 nm [32]. The absorption peak appeared at 413 nm in acidic conditions (pH = 6). Increasing the pH value of the reaction media, the SPR band of silver nanoparticles was shifted to lower wavelengths (402 nm at pH = 7) suggesting the formation of smaller in diameter AgNPs [33]. 

**Figure 2:**
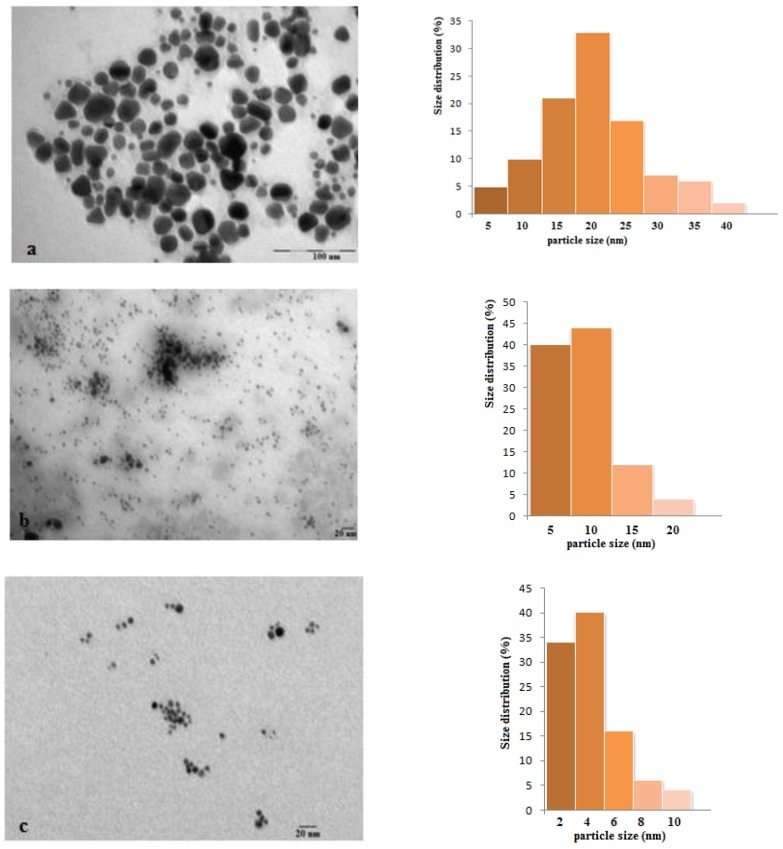

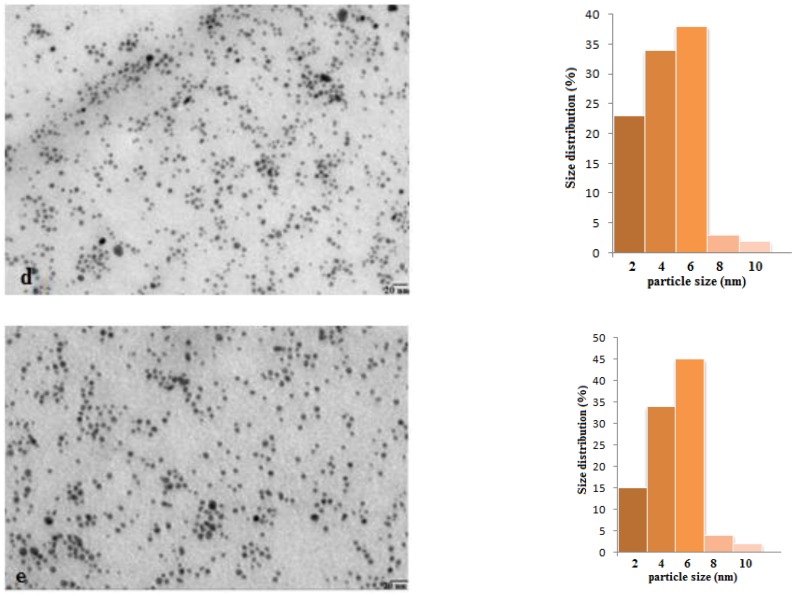
Transmission electron microscopy (TEM) images and corresponding histograms of silver nanoparticles at different pH values: (**a**) pH = 6; (**b**) pH = 7; (**c**) pH = 8; (**d**) pH = 9 and (**e**) pH = 10.

The size and shape of the phytosynthesized silver nanoparticles were monitored by TEM analysis. In acidic conditions, silver nanoparticles trend to aggregate whereas alkaline pH of the reaction medium led to formation of a large number of silver nanoparticles with smaller diameter. The TEM images (Figure 2) indicate the formation AgNPs, due to the stabilization by the biomolecules present in the *Ligustrum ovalifolium* L. fruit extract. In acidic conditions (pH = 6) the synthesized silver nanoparticles show irregular but mostly spherical shape morphology (Figure 2a) with sizes in the range of 25 nm. In order to realize the histograms for size determination of silver nanoparticles over 100 particles in random field of TEM images were measured. The particles obtained at pH = 7 are smaller in size (in the range of 10 nm Figure 2b) with more regular shape but they seem agglomerated. In alkaline conditions (pH = 8 to 10) the influence of the pH value on the size and morphology of the phytosynthesized silver nanoparticles was not significant (Figure 2c–e). The size of the spherical AgNPs obtained in these conditions was in the range of 6–13 nm with an average diameter of 7 nm (at pH = 10). Taking all these facts into account, the optimum pH value for the synthesis of *Ligustrum ovalifolium* fruit extract mediated silver nanoparticles is 10, value at which highly dispersed, spherical, regular in shape and small in diameter AgNPs were obtained.

**Figure 3:**
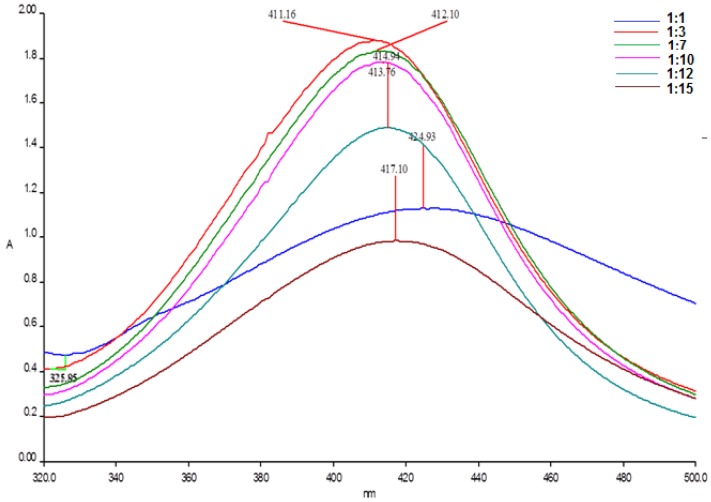
UV-Vis spectra of silver nanoparticles at different ratios fruit extract: silver nitrate solution.

The size, the shape and the yield of nanoparticles synthesis is also affected by the ratio of reducing agent to the metal ions. Various ratios: 1:1; 1:3; 1:7; 1:10; 1:12 and 1:15 respectively were investigated at the previously optimized pH value (pH = 10), in order to enhance the yield of AgNPs formation. The maximum absorbance in the UV-Vis spectra (Figure 3) was obtained for a 1:3 ratio fruit extract to silver nitrate solution. 

Moreover, the AgNPs obtained at this ratio exhibited surface plasmon resonance on a narrow absorption range, fact that indicates the monodispersity of the silver nanoparticles. By increasing the ratio to 1:15, the absorption maximum was shifted to a higher *λ*_max_ value, indicating a larger size of the formed nanoparticles and also a reduction in the absorption was observed, which may be due to aggregation of the AgNPs. The optimum ratio required for the synthesis of AgNPs with privet fruit extract was found to be 1:3. 

**Figure 4:**
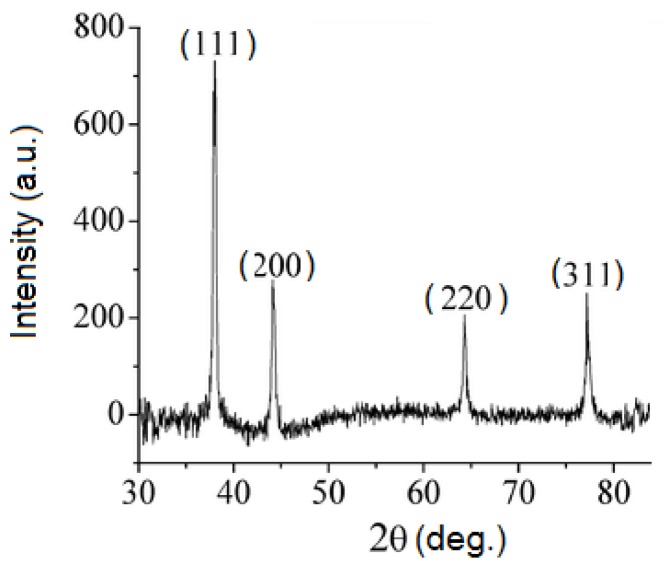
X-Ray Diffraction (XRD) pattern of silver nanoparticles.

The crystalline nature of the AgNPs was confirmed by the X-ray diffraction pattern (Figure 4). The X-ray diffractogram presents four prominent peaks at 2θ values of 37.59°, 44.07°, 64.17° and 77.22° corresponding to the (111), (200), (220) and (311) Bragg’s reflections assigned to lattice planes of face centred cubic (FCC) structure of metallic silver, in good agreement to reference of FCC structure of Joint Committee of Powder Diffraction Standard (JCPDS) file No. 04-0783. 

**Figure 5:**
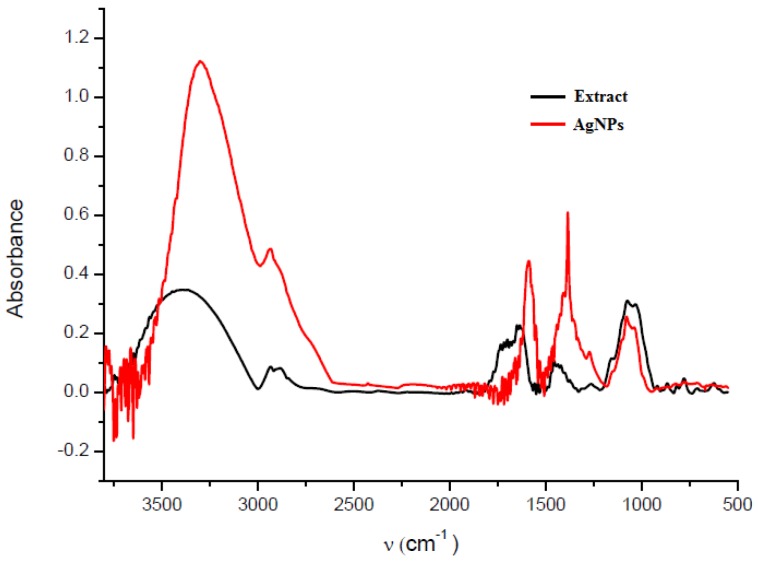
Fourier transform infrared (FTIR) spectra of fruit extract and silver nanoparticles.

Fourier transform infrared spectroscopy (FTIR) analysis was accomplished in order to identify the biomolecules from the fruit extract responsible for the reduction of silver ions and for the efficient stabilization of the silver nanoparticles. Figure 5 presents the FTIR spectra of the *Ligustrum ovalifolium* fruit extract and of the green synthesized AgNPs. The FTIR spectra of the fruit extract and silver nanoparticles present similar absorption bands. The FTIR analysis confirmed the dual role of the fruit extract as reducing and capping agent. The broad band at 3296 cm^−1^ is due to the O-H stretching vibration attributed to the polyphenols from the fruit extract. The FTIR spectrum of the silver nanoparticles shows several shifts of the absorption bands and also some intensity changes of these bands, compared to the spectrum of the fruit extract. The bands appearing at 2933, 1560 and 1383 and 1078 cm^−1^ indicate the presence of different functional groups and were assigned to C-H stretching vibration, C=C stretching vibration from aromatic rings and C-O stretching or O-H deformation vibration, respectively [34]. Most of the IR bands probably arise from the flavonoids anthocyanins and phenolic compounds from the fruit extract, indicating that the phytosynthesized AgNPs were stabilized by these biomolecules [35,36]. 

The observed peaks in the FTIR spectrum clearly indicate that the bioactive compounds from *Ligustrum ovalifolium* L. fruit extract are present as coating/capping agents at the surface of the silver nanoparticles. 

**Figure 6:**
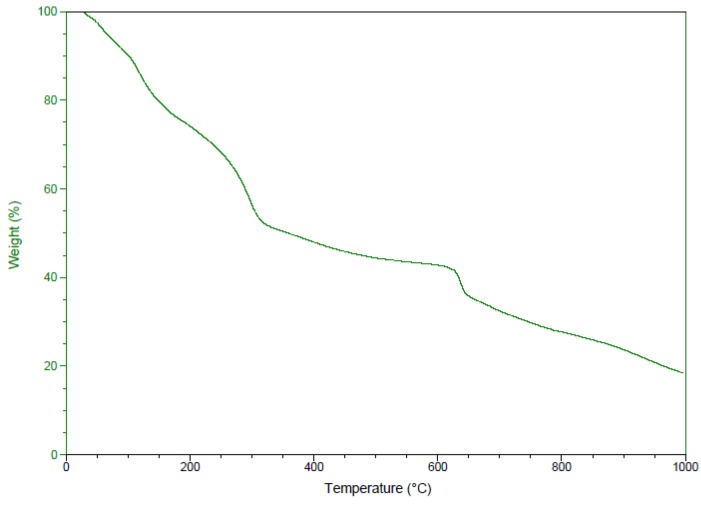
Thermogravimetric analysis (TGA) of silver nanoparticles.

The presence of the capping organic biomolecules on the surface of the privet extract synthesized silver nanoparticles was also confirmed by the thermogravimetric analysis (TGA). Figure 6 presents the TGA pattern of the green synthesized silver nanoparticles. The TGA curve clearly indicates an initial weight loss observed at around 100 °C, due to the loss of bound and unbound water molecules present in the obtained silver nanoparticles. A second weight loss appeared while temperature increases at 150–340 °C which accounted a 27% of the total AgNPs weight. This weight loss can be attributed to the degradation of organic compounds such as phenolic acids, flavonoids and carbohydrates [37]. A steady loss of weight appeared until 1000 °C which accounted for 29%, probably determined by the thermal degradation of resistant aromatic compounds. The observed behaviour in the TGA is a consequence of the thermal degradation of organic compounds from the fruit extract present in the nanoparticles powder which can be estimated to be 56% from the phytosynthesized AgNPs.

**Figure 7:**
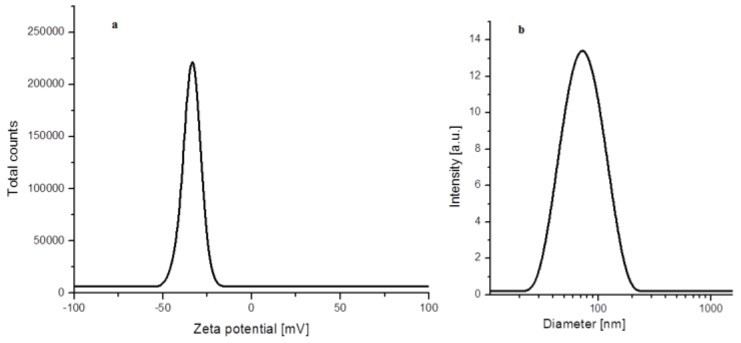
(**a**) Zeta potential. (**b**) hydrodynamic diameter of the obtained AgNPs.

The zeta potential of the green synthesized AgNPs was also determined using dynamic light scattering (DLS). The value of zeta potential gives important information about the surface of the silver nanoparticles and their stability. Generally, values of zeta potential of a colloidal suspension indicate the stability of the nanoparticles from solution [38]. Figure 7 presents the zeta potential (Figure 7a) and the DLS size distribution (Figure 7b) of the aqueous suspension of privet fruit extract mediated synthesized silver nanoparticles.

The value of the determined zeta potential was −31.8 mV, suggesting a high stability of the silver suspension. The negative value of the zeta potential once more confirms that the organic phytocomponents from the fruit extract, eventually with a phenolic structure, acted as capping agent and stabilized the AgNPs. The determined average particle hydrodynamic size was 91.8 nm, similar to that reported by other studies in which also phytosynthesized silver nanoparticles were obtained [39]. As expected, the particles size, in aqueous solution, obtained by DLS was larger than that obtained from TEM, due to the different principles used for analysis.

### 3.2. Cytotoxic Activity

The silver nanoparticles are known to inhibit cancer cells growth and thus could be potential anti-cancer therapeutic agents. Previous studies report the ability of AgNPs to inhibit growth of various cancer cell lines such as breast, ovarian, lung, glioblastoma, hepatic [40].

**Figure 8:**
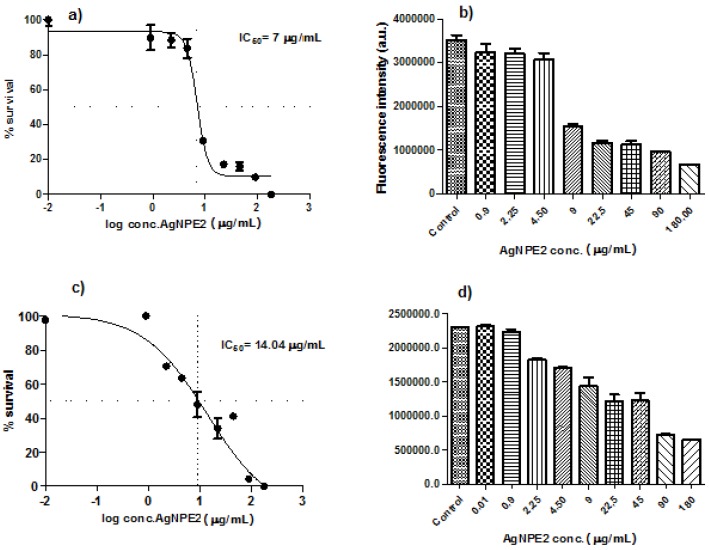
Dose-dependent toxicity of AgNPs on ovary carcinoma cell lines: (**a**) A2780; (**c**) A2780-Cis. The concentration of AgNPs for the significant reduction of cells viability: (**b**) A2780 (9 µg/mL); (**d**) A2780-Cis (2.25 µg/mL).

In vitro cytotoxicity of the biologically synthesized silver nanoparticles was investigated against two human ovarian cancer cell lines, A2780 and A2780Cis (cisplatin resistant) at different concentrations. The cells viability gradually decreased by increasing the concentration of AgNPs from 0.01 to 180 μg/mL (Figure 8) indicating a dose-dependent cytotoxic effect of the nanoparticles. IC50 values at 24 h incubation were calculated using nonlinear regression and four-parameter sigmoidal curve fit, with each point representing mean ±SEM in three separate measurements. The concentrations of the nanoparticles that reduced cell viability by 50% (IC50) were: 7 µg/mL for A2780 and 14.04 µg/mL for A2780-Cis, respectively. 

Our results are in agreement with those obtained in previous studies. Fahrenholtz et al. [41] determined IC50 value of cca. 7.2 μg/mL against A2780 cells when exposed to polyvinylpyrrolidone (PVP) coated silver nanoparticles but the advantage of using green synthesized nanoparticles could be the absence of a potential toxic synthetic capping agent. Young and co-workers [42] evaluated the influence of surface coating on the toxicity of silver nanoparticles in model organism *Caenorhabditis elegans*. They investigated citrate-coated, PVP-coated and gum arabic-coated AgNPs and demonstrated that the capping agent affects the degree of toxicity of silver nanoparticles. Gum arabic-coated (EC50 = 0.9 μM) have been proved to be 9-fold more toxic than PVP-coated (EC50 = 8 μM) which in turn were 3-fold more toxic than citrate-coated AgNPs (EC50 = 31 μM), all these differences being attributed to differences in toxicity of the surface coating agent. 

Lakshmanan and co-workers [43] also tested the cytotoxicity of phytosynthesized AgNPs on ovarian cancer cell lines but the obtained IC50 value was 30 μg/mL which is cca. 4-fold higher than the IC50 concentration of *Ligustrum ovalifolium* fruit extract mediated synthesized silver nanoparticles obtained in this study.

**Figure 9:**
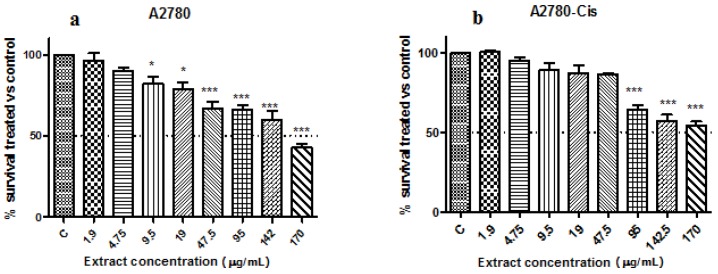
The viability of ovarian carcinoma cell lines treated with *Ligustrum ovalifolium* fruit extract: (**a**) A2780 and (**b**) A2780-Cis.

The extract showed low toxicity against the ovarian carcinoma cell lines, their viability being reduced significantly starting with the 95 µg/mL concentration for A2780 cells (*p* < 0.001) and 9.5 µg/mL (*p* < 0.5) and 47.5 (*p* < 0.001) for the cisplatin resistant cells (one-way ANOVA, Dunnett’s multiple comparisons test). The viability of both cell lines was reduced with 50% only when they were treated with the undiluted extract (no growth media) (Figure 9a,b). These results show that the extract has no/reduced toxicity towards the ovarian carcinoma cell lines.

The inhibitory effect on cancer cell growth enabled us to anticipate the promising anticancer potential of the obtained silver nanoparticles against ovarian cancer.

## 4. Conclusions

The present study presents for the first time an eco-friendly, cost effective, efficient and simple method for the biosynthesis of AgNPs using bioactive compounds from the aqueous extract of *Ligustrum ovalifolium* L. fruits as reducing and capping agents. The synthesized nanoparticles proved potential cytotoxic effect against ovarian carcinoma cells and may be suitable for biomedical applications as promising alternative therapeutic agents for cancer.

## References

[1] Janardhanan R., Karuppaiah M., Hebalkar N., Rao T.N. (2009). Synthesis and surface chemistry of nano silver particles. Polyhedron.

[2] Thakkar N., Mhatre S.S., Parich R.Y. (2010). Biological synthesis of metallic nanoparticles. Nanomed. Nanotechnol. Biol. Med..

[3] Wang Z.H., Hsu C.C., Yin M.C. (2009). Antioxidative characteristics of aqueous and ethanol extracts of glossy privet fruit. Food Chem..

[4] Moldovan B., David L., Vulcu A., Olenic L., Perde-Schrepler M., Fischer-Fodor E., Baldea I., Clichici S., Filip G.A. (2017). In vitro and in vivo anti-inflammatory properties of green synthesized silver nanoparticles using *Viburnum opulus* L. fruit extract. Mater. Sci. Eng. C.

[5] Opris R., Tatomir C., Olteanu D., Moldovan R., Moldovan B., David L., Nagy L., Decea N., Kiss M., Filip G.A. (2017). The effect of *Sambucus nigra* L. extract and phytosynthesized gold nanoparticles on diabetic rats. Colloids Surf. B.

[6] Mohammed A.E., Al-Qahtani A., al-Mutairi A., Al-Shamri B., Aabed K. (2018). Antibacterial and cytotoxic potential of biosynthesized silver nanoparticles by some plant extracts. Nanomaterials.

[7] Moldovan B., David L., Achim M., Clichici S., Filip G.A. (2016). A green approach to phytomediated synthesis of silver nanoparticles using *Sambucus nigra* L. fruit extract and their antioxidant activity. J. Mol. Liq..

[8] Jayaprakash N., Vijaya J.J., Kaviyarasu K., Kombaiah K., Kennedy L.J., Ramalingam R.J., Munusamy M.A., Al-Lohedan H.A. (2017). Green synthesis of Ag nanoparticles using Tamarind fruit extract for the antibacterial studies. J. Photochem. Photobiol. B Biol..

[9] Dong C., Cao C., Zhang X., Zhan Y., Wang X., Yang X., Zhou K., Xiao X., Yuan B. (2017). Woolfberry fruit (*Licium barbarum*) extract mediated novel route for the green synthesis of silver nanoparticles. Opt. Int. J. Light Electron Opt..

[10] Filip A.G., Potara M., Florea A., Baldea I., Olteanu D., Bolfa P., Clichici S., David L., Moldovan B., Olenic L. (2015). Comparative evaluation by scanning confocal Raman spectroscopy and transmission electron microscopy of therapeutic effects of noble metal nanoparticles in experimental acute inflammation. RSC Adv..

[11] Moldovan B., Filip A., Clichici S., Suharovschi S., Bolfa P., David L. (2016). Antioxidant Activity of Cornelian Cherry (*Cornus mas* L.) Fruits Extracts and the in Vivo Evaluation of their Anti-inflammatory Effects. J. Func. Foods.

[12] Danila O.O., Berghian A.S., Dionisie V., Gheban D., Olteanu D., Tabaran F., Baldea I., Katona G., Moldovan B., Clichici S. (2017). The effects of silver nanoparticles on behaviour, apoptosis and nitro-oxidative stress in offspring Wistar rats. Nanomedicine.

[13] Ajan R., Chandran K., Harper S.L., Yun S.-I., Kalaichelvan P.T. (2015). Plant extract synthesized silver nanoparticles: An ongoing source of novel biocompatible materials. Ind. Crop. Prod..

[14] Prabhu S., Poulose E.K. (2012). Silver nanoparticles: mechanism of antimicrobial action, synthesis, medical applications and toxicity effects. Int. Nano Lett..

[15] Singh P., Kin Y.J., Zhang D., Yan D.C. (2016). Biological synthesis of nanoparticles from plants and microorganisms. Trends Biotechnol..

[16] Wei L., Lu J., Xu H., Patel H., Chen Z.S., Chen G. (2015). Silver nanoparticles: synthesis, properties and therapeutic applications. Drug Discov. Today.

[17] Garcia-Barrasa J., Lopez-de-Luzuriaga J.M., Monge M. (2011). Silver nanoparticles: synthesis through chemical methods in solution and biological applications. Cent. Eur. J. Chem..

[18] Sharma V.K., Yngard R.A., Lin Y. (2009). Silver nanoparticles: green synthesis and their antimicrobial activities. Adv. Colloid Interface Sci..

[19] Wang J., Shan A., Liu T., Zhang C., Zhang Z. (2012). In vitro immunomodulatory effects of an oleanolic acid-enriched extract of *Ligustrum lucidum* fruit (*Ligustrum lucidum* supercritical CO_2_ extract) on piglet immunocytes. Int. Immunopharmacol..

[20] Yin T.K., Wu W.K., Pak W.F., Ko K.M. (2001). Hepatoprotective action of an oleanolic acid-enriched extract of Ligustrum lucidum fruits is mediated through an enhancement on hepatic glutathione regeneration capacity in mice. Phytother. Res..

[21] Lee S.I., Oh S.H., Park K.Y., Park B.H., Kim J.S., Kim S.D. (2009). Antihyperglycemic effects of fruits of privet (*Ligustrum obtusifolium*) in streptozotocin induced diabetic rats fed a high fat diet. J. Med. Food.

[22] Lin H.M., Yen F.L., Ng L.T., Lin C.C. (2007). Protective effects of *Ligustrum lucidum* fruit extract on acute butylated hydroxytoluene-induced oxidative stress in rats. J. Ethnopharmacol..

[23] Jeong J.C., Kim J.W., Kwong C.H., Kim T.H., Kim Y.K. (2011). *Fructus ligustri lucidi* extracts induce human glioma cells death through regulation of Akt/mTOR pathway in vitro and reduce glioma tumor browth in U87MG xenograft mouse model. Phytother. Res..

[24] Zhang J.F., He M.L., Dong Q., Xie W.D., Chen Y.C., Lim M.C., Leung P.C., Zhang Y.O., Kung H.F. (2011). Aqueous extract of fructus Ligustri lucidi enhances the sensitivy of human colorectal carcinoma DLD-1 cells to doxorubicin-induced apoptosis via Tbx3 suppression. Integr. Cancer Ther..

[25] Hu B., Du Q., Deng S., An H.-M., Pan C.-H., Shen K.-P., Xu L., Wei M.-M., Wang S.-S. (2014). *Ligustrum lucidum* Ait. fruit extract induces apoptosis and cell senescence in human hepatocellular carcinoma cells through upregulation of p21. Oncol. Rep..

[26] Zeisser-Labouebe M., Lange N., Gurniy R., Delie F. (2006). Hypericin-loaded nanoparticles for the photodynamic treatment of ovarian cancer. Int. J. Pharm..

[27] Iravani S., Korbekandi H., Mirmohammadi S.V., Zolfaghari B. (2014). Synthesis of silver nanoparticles: Chemical, physical and biological methods. Res. Pharm. Sci..

[28] Singleton V.L., Orthofer R., Lamuela-Raventós R.M. (1999). Analysis of total phenols and other oxidation substrates and antioxidants by means of Folin-Ciocalteu reagent. Met. Enzymol..

[29] Arnao M.B., Cano A., Acosta M. (2001). The hydrophilic and lipophilic contribution to total antioxidant activity. Food Chem..

[30] Kiss A.K., Mank M., Melzig M.F. (2008). Dual inhibition of metallopeptidases ACE and NEP by extracts and iridioids from *Ligustrum vulgare* L.. J. Ethnopharmacol..

[31] Poopathi S.H., De Britto L.J., Praba V.L., Mani C., Praveen M. (2015). Synthesis of silver nanoparticles from *Azadirachta indica*—A most effective method for mosquito control. Environ. Sci. Pollut. Res..

[32] Ashokkumar S., Ravi S., Kathiravan V., Velmurugan S. (2014). Rapid biological synthesis of silver nanoparticles using *Leucas martinicensis* leaf extract for catalytic and antibacterial activity. Environ. Sci. Pollut. Res. Sci..

[33] Sanchez G.R., Castilla C.L., Gomez N.B., Garcia A., Marcos R., Carmona E.R. (2016). Leaf extract from the endemic plant *Peumus boldus* as an effective bioproduct for the green synthesis of silver nanoparticles. Mater. Lett..

[34] Elemike E.E., Onwudiwe D.C., Mkhize Z. (2016). Eco-friendly synthesis of AgNPs using *Verbascum thapsus* extract and its photocatalytic activity. Mater. Lett..

[35] He Y., Li X., Wang J., Yang Q., Yao B., Zhao Y., Zhao A., Sun W., Zhang Q. (2017). Synthesis, characterization and evaluation cytotoxic activity of silver nanoparticles synthesized by Chinese herbal *Cornus officinalis* via environment friendly approach. Environ. Toxicol. Pharmacol..

[36] Dhand V., Soumya L., Bharadwaj S., Chakra S., Bhatt D., Sreedhar B. (2016). Green synthesis of silver nanoparticles using *Coffea Arabica* seed extract and its antibacterial activity. Mater. Sci. Eng. C.

[37] Sun Q., Cai X., Li J., Zheng M., Chen Z., Yu C.-P. (2014). Green synthesis of silver nanoparticles using tea leaf extract and evaluation of their stability and antibacterial activity. Colloids Surf. A Physicochem. Eng. Asp..

[38] El Badawy A.M., Luxton T.P., Silva R.G., Scheckel K.G., Suidan M.T., Tolaymat T.M. (2010). Impact of environmental conditions (pH, ionic strength and electrolyte type) on the surface charge and aggregation of silver nanoparticles suspensions. Environ. Sci. Technol..

[39] Gengan R.M., Anand K., Phulukdaree A., Chuturgoon A. (2013). A549 cell line activity of biosynthesized silver nanoparticles using Albizia adianthifolia leaf. Colloids Surf. B.

[40] Ahmed K.B.R., Nagy A., Brown R.P., Zhang Q., Malhan S.G., Goering P.L. (2017). Silver nanoparticles: significance of physico-chemical properties and assay interference on the interpretation of in vitro cytotoxicity studies. Toxicol. Vitr..

[41] Fahrenholtz C.D., Swanner J., Ramirez-Perez M., Singh R.N. (2017). Heterogeneous response of ovarian cancer cells to silver nanoparticles as a single agent and in combination with cisplatin. J. Nanomater..

[42] Young X., Gondikas A.P., Marinakos S.M., Auffan M., Liu J., Hsu-Kim H., Meyer J.N. (2012). Mechanism of silver nanoparticle toxicity is dependent on dissolved silver and surface coating in *Caenorhabditis elegans*. Environ. Sci. Technol..

[43] Lakshmanan G., Sathiyaseelan A., Kalaichelvan P.T., Murugesan K. (2018). Plant mediated synthesis of silver nanoparticles using fruit extract of *Cleome viscosa L*.: Assessment of their antibacterial and anticancer activity. Karbala Int. J. Mod. Sci..

